# Cancer vaccines: past, present and future; a review article

**DOI:** 10.1007/s12672-022-00491-4

**Published:** 2022-05-16

**Authors:** Eddie Grimmett, Bayan Al-Share, Mohamad Basem Alkassab, Ryan Weng Zhou, Advait Desai, Mir Munir A. Rahim, Indryas Woldie

**Affiliations:** 1 Department of Biomedical Sciences, University of Windsor, Windsor, ON Canada; 2 Barbara Ann Karmanos Cancer Institute, Detroit, MI USA

## Abstract

Immunotherapy and vaccines have revolutionized disease treatment and prevention. Vaccines against infectious diseases have been in use for several decades. In contrast, only few cancer vaccines have been approved for human use. These include preventative vaccines against infectious agents associated with cancers, and therapeutic vaccines used as immunotherapy agents to treat cancers. Challenges in developing cancer vaccines include heterogeneity within and between cancer types, screening and identification of appropriate tumour-specific antigens, and the choice of vaccine delivery platforms. Recent advances in all of these areas and the lessons learnt from COVID-19 vaccines have significantly boosted interest in cancer vaccines. Further advances in these areas are expected to facilitate development of effective novel cancer vaccines. In this review, we aim to discuss the past, the present, and the future of cancer vaccines.

## Introduction

Immunization has been practiced for hundreds of years starting with the use of snake venom to protect from snakebite to the development of smallpox vaccine in 1978. However, few vaccines have been effective in reducing cancer incidences in a population and in treating cancers. The past few years have seen tremendous breakthrough in vaccine technology, including the most recent use of nucleic acid vaccines against COVID-19 infection, and the development of cancer immunotherapy. Further advances in cancer vaccine technology are predicted in the future, which are discussed in this review.

## A brief overview of the immune system

A full description of the immune system is beyond the scope of this review article. However, to better understand the concepts of immunotherapy and vaccines, we provide a brief overview of the immune system and its association with cancer immunity. The immune system is an integrated system of soluble molecules, cells, tissues, and organs that is capable of recognizing an invading antigen and initiating a cascade of responses that ultimately lead to the elimination of the foreign antigen. The ability to distinguish foreign versus self-antigens is a hallmark feature of the immune system. Based on the recognition mechanisms, the effector cells involved, and the speed at which effector mechanisms are elicited, immune responses can be broadly categorized into innate immunity and adaptive immunity. Innate immunity is present at birth and provides a generalized immediate response to foreign invaders [1]. The effector cells of the innate immune system including natural killer (NK) cells, neutrophils, macrophages, etc. Cells of the innate immune system recognize invading pathogens and cells with the help of germ-line encoded pattern recognition receptors (PRR) that recognize pathogen-associated molecular patterns (PAMP), which are usually shared among many different types of pathogens [1]. Adaptive immunity, on the other hand, develops after birth and is antigen specific [2]. Antigen-specific recognition is mediated by receptors, which are generated through gene recombination, giving rise to receptor diversity and antigen-specificity [2]. Adaptive immunity is mediated by B and T lymphocytes: the latter are either CD8^+^ cytotoxic and CD4^+^ helper T cells [3]. B cells work by producing antibodies (humoral immunity) against foreign antigens aiming to block their impact on cells and tissues, while T cells recognize and eliminate diseased cells (cellular immunity) [3]. An effective immune response against an antigen involves a concerted effort by both the innate and the adaptive arms of the immune system. Soluble factors, such as cytokines and chemokines, produced by the cells of the innate immune system are essential for activation of other immune cells, including B and T cells of the adaptive immune system. As well, processing of antigens by antigen presenting cells (APC), such as dendritic cells (DC), and presentation of antigenic peptides on human leukocyte antigens (HLA), the major histocompatibility complex (MHC) in humans, are critical for induction of adaptive immunity [4]. T cells possess antigen-specific T cell receptors (TCR), which recognize antigens presented on MHC molecules. A hallmark feature of the adaptive immune system, which makes long term immunity and immunization by vaccination possible, is antigen-specific immunological memory. After a primary challenge with an antigen, the adaptive immune system can mount a better, stronger, and faster response against the same antigen upon subsequence exposures [2]. Vaccines work by exposing the immune system to an immunizing antigen, which is derived from a pathogen, in the absence of an infection. Subsequently, the adaptive immunity and immunological memory elicited against the immunizing antigen protects an immunized individual against the pathogen from which the antigen was derived. Advances in understanding how the immune system recognizes and responds against pathogens and non-self/foreign cell have made possible the development of vaccines and other immune-based therapies.

In addition to immunity against foreign pathogens, several lines of evidence have demonstrated the important role of the immune system in cancer immunosurveillance and in immunity against cancers. These include: (i) increased incidences of cancers in individuals with a compromised immune system, (ii) increased incidences of cancers in patients undergoing immunosuppressive therapy, (iii) infiltration of immune cells into the tumour microenvironment and their association with better cancer prognosis, and (iv) presence of tumour antigen-specific T cells in cancer patients [5–8]. Not only the immune system responds against and eliminates cancers, but in doing so, it also shapes the tumour cells and the tumour microenvironment in a way that makes tumours resistant to further immune assault. This process is termed tumour immunoediting [9]. Tumours are also equipped with a multifaceted immune escape mechanism to evade detection and elimination by the immune system [10]. Over the past decades, advances made in understanding cancer immunosurveillance, immunoediting, immune escape processes have resulted in the development of affective cancer immunotherapies and vaccines, and will inform future development of novel immunotherapies and novel cancer vaccines.

## Cancer immunotherapy

Although it is still far from being fully conquered, cancer treatment has transformed through the years. Surgery was the only treatment option prior to the development of chemotherapy, which was used for the first time in 1942 at Yale. The remarkable history of William Coley (Bone Surgeon, New York 1890–1936) in pioneering cancer immunotherapy, long before cancer immunosurveillance theory was established, is worth mentioning. Following his observation, in the late 1890s, of possible anti-cancer effect from bacteria infection, he started treating Osteosarcoma patients with a heat killed bacteria preparation (Coley’s toxin), which occasionally resulted in tumor regression [11]. Cancer immunotherapy has evolved since the pioneering work of William Coley. In the past 20 years, we have witnessed a significant breakthrough in cancer treatment with the addition of more targeted therapies and immunotherapy [12]. Cancer cells can avoid recognition by the immune system, and the immunosuppressive nature of the tumour micro-environment renders tumour-infiltrating lymphocytes ineffective at tumour elimination in vivo [10, 13]. The goal of immunotherapy is to overcome immune suppression in the tumour microenvironment and use the natural ability of the immune system to attack and eliminate cancers.

Cancer immunotherapy has come a long way from immune surveillance hypothesis entertained by Paul Ehrlich in 1909, to the development of various immunotherapy medications that are currently in use [14]. The scope of cancer immunotherapy grew from the use of immune stimulating cytokines like interleukin-2 (IL-2) and Interferon alpha (IFNα) in renal cell carcinoma and melanoma, to the use of immune modulating antibodies against Cytotoxic T cell Lymphoma Antigen 4 (CTLA 4) and Programmed Death 1 (PD-1) molecules. Although response rate was low (10–15%) and toxicity was significant, high dose IL-2 offered durable remission for the few responding patients [15].

Discovered by Pierre Goldstein in 1987, CTLA-4 is expressed on T cells after activation and its physiologic role is to suppress T cell responses. It is also referred to as a brake to the immune system or immune checkpoint. Following the observation of tumour shrinkage in mice treated with CTLA-4 antibodies, the monoclonal antibody to CTLA-4, Ipilimumab was developed for human use [16]. Ipilimumab was approved by the FDA in 2011 for the treatment of unresectable or metastatic melanoma. The approval was based on the phase III double blind study that showed overall response rate of 10.9% with 60% maintaining the response for at least 2 years. Immune related toxicity was seen in 60% of the patients with 7 deaths attributed to immune related toxicity [17]. Although this was a great breakthrough, the low response rate, albeit durable, and high rate of immune related toxicity (20% grade III and IV) necessitated the need for safer and more effective immunotherapy drugs. The anti-CTLA-4 monoclonal antibody therapy is still used in combination with other immunotherapy drugs in patients with metastatic melanoma [18, 19].

PD-1 is another immune checkpoint receptor expressed on activated T cells. Following the discovery of PD-1 and its ligand PD-L1 by Tasuku Honjo and Lieping Chen, respectively, it was shown (by Lieping Chen) that PD-L1 was upregulated in several cancers and blocking of the PD-L1/PD-1 interactions lead to tumour regression in mice [20]. This discovery led to development of monoclonal antibodies targeting PD-1/PD-L1 interactions to treat several types of human cancers, including renal cell carcinoma, melanoma, Hodgkin’s lymphoma, non-small cell lung cancer and others. For instance, Nivolumab, a PD-1 monoclonal antibody is approved by the FDA for the treatment of advanced melanoma (Dec 22, 2014), metastatic renal cell carcinoma (Nov 23, 2015), Hodgkin’s lymphoma (May 17, 2016), metastatic urothelial carcinoma (Feb 2, 2017) among others [21]. Pembrolizumab, another PD-1 monoclonal antibody is FDA approved for the treatment of advanced melanoma (Sep 4, 2014), advanced non-small cell lung cancer (Oct 2, 2015), head and neck squamous cell carcinoma (Aug 5, 2016), classical Hodgkin’s lymphoma (Mar 15, 2017), and advanced renal cell carcinoma (Aug 11, 2021) among others [22].

Another fascinating progress in utilizing the immune system to eradicate cancer is the use of chimeric antigen receptor (CAR) T cell therapy. CAR is a genetically modified receptor that is specific for a tumour-associated antigen (TAA). Patient T cells are transfected with a viral vector containing the CAR genetic code to produce CAR T cells, which are then adoptively transferred to the patient. CAR T cells are potent immune effectors that will expand and form a long-term tumour antigen-specific immunological memory. Many versions of the CAR T cell have been developed over the years to achieve a better anti-tumour activity, proliferation capacity, and improved in vivo persistence [23]. Currently, CAR T cell therapy is FDA approved for use in a variety of hematologic malignancies, including acute lymphoblastic leukemia and diffuse large B cell lymphoma [24].

Despite all these success stories, cancer immunotherapy is still not curative. Further research is needed to make cancer immunotherapy more effective and safer. Based on the tremendous achievements and the progress made so far in understanding anti-cancer immune responses, it is inevitable to anticipate growing interest to further exploit the interaction between cancer and the immune system to develop additional therapeutic agents, including cancer vaccines.

## The current state of cancer vaccines

**Fig. 1:**
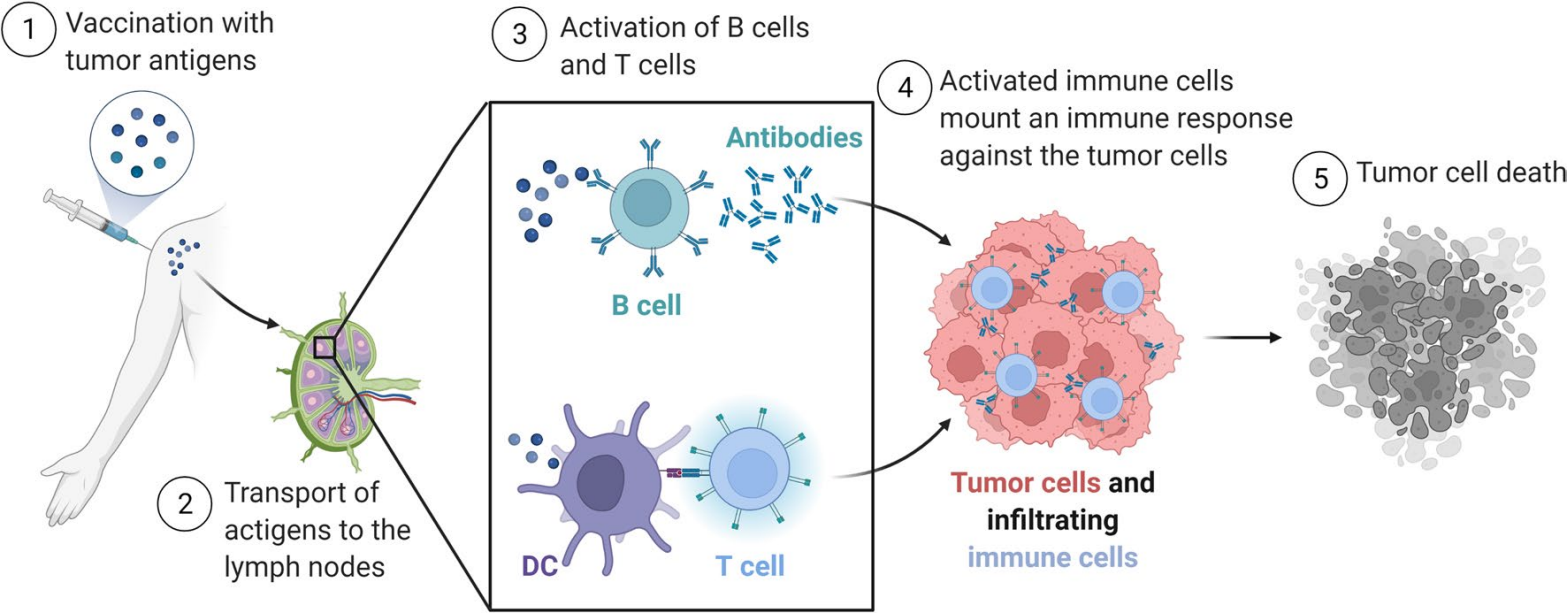
Cancer vaccine. After vaccination, tumour antigens are carried to the lymph nodes, where they activate antigen-specific B and T cells. B cells recognize antigens directly, while T cells are activated by dendritic cells (DC) which process and present antigen on MHC molecules. Antibodies produced by activated B cells and activated effector T cells infiltrate tumours and induce tumour cell death

Cancer vaccines are designed with the intent of inducing an immune response against tumour antigens (Fig. 1). Despite decades of research and development, only few cancer vaccines have been approved for human use (Table 1): several other vaccines are in various phases of clinical trial (described below). The success of these cancer vaccines depends on several factors, including the type of antigens used, the tumour microenvironment, the immune landscape of the tumour, and the different vaccine formulations.

**Table 1:** The approved vaccines for cancer prevention and therapy

Vaccine	Target antigen	Use	Cancer Type
Hepatitis B	Hepatitis B virus (HBV) surface antigen (HBsAg)	Preventative	Hepatocellular carcinoma caused by chronic HBV infection
Cervarix	L1 protein of Human papilloma virus (HPV) types 16 and 18	Preventative	HPV-associated cervical, oropharyngeal, anal, penile, and vulvovaginal cancers
Gardasil-4	L1 protein of HPV types 6, 11, 16, and 18	Preventative	HPV-associated cervical, oropharyngeal, anal, penile, and vulvovaginal cancers
Gardasil-9	L1 protein of HPV types 6, 11, 16, 18, 31, 33, 45, 52, and 58	Preventative	HPV-associated cervical, oropharyngeal, anal, penile, and vulvovaginal cancers
Bacillus Calmette-Guerin (BCG)	Non-pathogenic *Mycobaterium bovis*	Therapeutic	high-risk non-muscle-invasive bladder cancer (NMIBC)
Sipuleucel-T (Provenge)	Prostate acid phosphatase (PAP) protein	Therapeutic	Castration-resistant prostatic cancer

### Preventative cancer vaccines

Among the first effective vaccines in preventing cancers were those targeting viral infections associated with cancer development. Hepatitis B virus (HBV) is a leading cause of chronic liver disease that increases risk of hepatocellular carcinoma (HCC) [25, 26]. The vaccine for HBV has been available since the early 1980s and is recommended by World Health Organization (WHO) for infants soon after birth. Three doses of the vaccine are highly affective in providing long-lasting immunity against chronic HBV infection [25]. It was the first preventative vaccine shown to reduce incidence of HCC in vaccinated individuals [27]. Taiwan was one of the first nations to implement a nation-wide HBV vaccination program, initially given to infants born to infected mothers, and later extended to all infants in 1984. Subsequent studies showed a significant drop in HCC incidence in vaccinated Taiwanese children up to 20 years after the implementation of this vaccination program [27, 28]. Similar studies in Thailand, where the national neonate HBV vaccination program was implemented in the late 1980s, showed significantly lower HCC incidents in children vaccinated at birth [29]. A more impressive success story comes from the United States, where universal newborn immunization with HBV vaccine implemented in 1984 in Alaska Natives, has eliminated HCC among Alaska Native children under the age of 20 years [30]. Human papilloma virus (HPV) is a sexually transmitted virus associated with a few cancers, including cervical, oropharyngeal, anal, penile, and vulvovaginal cancers [31]. HPV vaccines are available since 2006 and is recommended as prophylactic vaccine for females and males over the age of 11 years and before the onset of sexual activity, i.e., before exposure to the virus [32]. Three HPV vaccines have been approved for prevention of HPV-related diseases in humans. HPV vaccines provide immunity against high-risk HPV types. These vaccines include the bivalent vaccine (Cervarix) against HPV types 16 and 18, the quadrivalent vaccine (Gardasil-4) against HPV types 6, 11, 16, and 18, and the nonavalent vaccine (Gardasil-9) against HPV types 6, 11, 16, 18, 31, 33, 45 52, and 58 [32]. HPV vaccines are safe, highly immunogenic in adolescence, and induce long-lasting antibody response and protection into adulthood [33]. Several phase II/III clinical trials have demonstrated high efficacy of HPV vaccines in reducing the risk of HPV-related high-grade cervical, vulvar, vaginal lesions, and genital warts in females [34–38]. A follow up monitoring of HPV vaccination programs in the United States and Australia targeting females aged between 11 and 26 years revealed significantly reduced HPV-related cervical lesions and abnormalities in the vaccinated compared to unvaccinated women [36, 39]. In the long term, high coverage HPV vaccination programs are expected to substantially reduce the rate of HPV-related cancers in both males and females.

No preventative vaccine for non-viral cancers has yet been approved for use in humans. This is partly due to the unavailability of appropriate TAA and the risk of cross-reaction with self-molecules on healthy tissues causing autoimmunity. However, a few TAAs have now been safely used in therapeutic vaccine trials without substantial autoimmune effects. Moreover, presence of autoantibodies against TAAs is associated with better prognosis in cancer patients and could even reduce cancer risk [40–42], suggesting that prophylactic induction of anti-TAA immune responses in the absence of cancer could potentially reduce cancer incidence. Additionally, advances in clinical imaging and diagnostic tools have improved the early detection of cancers and pre-malignant lesions, which provides an early window for the use of protective vaccines that induces anti-cancer immunity prior to its development.

### Therapeutic cancer vaccines

Therapeutic cancer vaccines are used as immunotherapeutic tools to treat an active disease. Only two therapeutic vaccines have been approved in cancer immunotherapy. These include Bacillus Calmette-Guerin (BCG) vaccine for treatment of early-stage bladder cancer, and Sipuleucel-T (Provenge), a dendritic cell (DC)-based vaccine for treatment of castration-resistant prostate cancer [43, 44].

BCG is a non-pathogenic bacterium derived from *Mycobacterium bovis*, which induces a protective immune response against tuberculosis caused by *M*. *tuberculosis*. It remains the only commercially available vaccine against tuberculosis [45]. The use of BCG for treatment of high-risk non-muscle-invasive bladder cancer (NMIBC) was approved after it was shown in the mid-late 1970s that intravesical instillation of this bacterium could halt disease progression and recurrence of NMIBC [46, 47]. BCG vaccine has since been routinely used for the treatment of NMIBC, however, the precise immunological mechanisms of BCG therapy in NMIBC is unclear. The treatment consists of a weekly instillation of BCG into the bladder for 6 weeks after resection of the tumours. Patients can then enter a phase of maintenance treatment, which consists of weekly instillation of BCG vaccine into the bladder for 3–6 weeks every 3 months for 1–3 years [44]. BCG therapy is associated with some complications in the genitourinary tract, including cystitis, bladder ulceration, penile lesions, prostatitis, and kidney infection, as well as systemic complications, such as fever, disseminated infections, BCG sepsis, etc.[48].

Sipuleucel-T vaccine is an DC-based vaccine, which uses autologous DC to stimulate cellular immune responses mediated by T cells against prostatic acid phosphatase (PAP) in castration-resistant prostate cancer patients. DCs are antigen presenting cells that can efficiently induce antigen-specific priming and activation of T cell [4]. They express class I and class II HLA molecules and present processed antigenic peptide: HLA complexes to T cells. Sipuleucel-T vaccine is prepared by incubating patient DCs with a fusion protein, consisting of PAP linked to granulocyte macrophage colony-stimulating factor (GM-CSF), to induce DC activation, processing of PAP antigenic epitopes, and expression of antigenic peptide: HLA complexes and costimulatory molecules. Activated DCs are then reinfused into the patient, which will present antigens and activate T cell responses against PAP protein [49–51]. Sipuleucel-T vaccine was approved for treatment of castration-resistant prostate cancer after phase III trails showed significantly improved median survival and decreased risk of death in patients receiving the vaccine compared to the placebo-treated group, most notable in patients with a Gleason score of 7 or less [49, 52, 53]. The treatment consists of three infusions of approximately 50 million autologous DCs given every two weeks. Adverse reactions were found to be mild in most patients and included flu-like symptoms, back pain, joint pain, muscle aches, headache, vomiting, constipation, diarrhea, anemia, and dizziness [43].

### Cancer vaccines in clinical trials

In addition to the vaccines approved for treatment of cancers, several other cancer vaccines are in various phases of clinical trial, some of which are described here. A complete list of clinical trials involving cancer vaccines can be found at *clinicaltrials.gov*. Amongst these, are the whole tumour cell vaccines, which use killed tumour cells to stimulate anti-cancer immune responses. The advantage of using killed tumour cells as vaccine is that they can induce immune responses against multiple tumour antigens, which do not have to be prospectively identified. Tumour cells can also be genetically modified to secret immunomodulatory cytokines, such as GM-CSF, which can promote DC activation, and enhance antigen presentation and activation of adaptive immune responses [54]. Both autologous and allogeneic tumour cell vaccines (e.g. GVAX and Vigil vaccines) engineered to produce and release GM-CSF are under investigation and have been studied as monotherapy or in combination with other immunotherapy agents in cancers including pancreatic, prostate, ovarian and colon cancer [55–60]. A randomized trial that tested Vigil autologous GM-CSF vaccine in the frontline maintenance for stage III-IV ovarian cancer showed relapse-free survival (RFS) clinical benefit, specifically in patients without BRCA1 and BRCA2 mutations [55]. Despite, inducing immune responses in patients in several trials, whole tumour cells vaccines have so far failed to show a significant therapeutic efficacy to be considered for cancer immunotherapy. Additionally, GM-CSF can induce recruitment of myeloid suppressor cells, which could adversely affect anti-cancer immune responses in the tumour microenvironment [58]. Never the less, the relative simplicity of the procedure and the range of tumour antigens provided by whole tumour vaccines make them an attractive immunotherapeutic agent. Further trials in the future are aimed at increasing immunogenicity of whole tumour vaccines to elicit protective immunity and enhanced therapeutic efficacy against cancers.

Anti-idiotypic (anti-id) vaccines, such as Racotumumab, have shown some therapeutic efficacy and have been approved in Cuba and Argentina for treatment of advanced or recurrent non-small cell lung cancer (NSCLC). Anti-id vaccines use monoclonal antibodies that bind to antibodies specific to tumour antigens and block their interactions. Therefore, anti-id antibodies can mimic the structure of tumour antigens and elicit antigen-specific immune responses. Anti-id vaccines can be used to elicit immune responses against carbohydrate and lipid antigens, which are less immunogenic than protein antigens. Racotumumab induced immune responses against Neu-glycolyl-containing gangliosides, sulfatides, and other antigens expressed in tumours [61, 62]. Preclinical trials have demonstrated a strong antimetastatic effect, and clinical data have demonstrated its safety in NSCLC, breast cancer, advanced melanoma, and a significant clinical benefit in NSCLC patients compared to best supportive care [62–65].

Immunogenicity of tumour antigens and high mutational load are essential for response to immunotherapy. Glioblastoma, for instance, has low mutational load: hence, the limited intratumoral immune cell infiltration [66]. A number of different vaccine approaches including peptide vaccines and cell-based vaccines (DC and tumour-cell vaccines) have been trialed for treatment of glioblastoma, however, results of these studies have not been satisfactory to be considered for treatment [67–69]. In a phase I trial, glioma actively personalized vaccine (GAPVAC) using peptide antigens with polyriboinosinic-polyribocytidylic acid-poly-L-lysine carboxymethylcellulose (poly-ICLC) and GM-CSF adjuvants showed feasibility and a favourable safety with strong immunogenicity in newly diagnosed glioblastoma [70]. In this trial, patients were treated with a vaccine derived from unmutated premanufactured antigens (APVAC1) followed by a vaccine targeting mutated neoepitopes (APVAC2). APVAC1 induced a sustained response of memory CD8^+^ T cells, while APVAC2 mainly induced CD4^+^ T helper 1 type responses against predicted neoepitopes [70].

In many of the trials noted above [56, 64, 69, 70], cancer vaccines are tried in conjunction with approved immunotherapies or other standard of care therapies. While vaccines can induce activation of anti-cancer immune effector functions, the activated immune cells must contend with the highly immunosuppressive microenvironment encountered within some tumors. The immunosuppressive mechanisms within tumor include, barriers to immune infiltration by anti-cancer immune cells, selective recruitment of cells with immunosuppressive activity, induction of T cell death, production of enzymes and metabolites that are immunosuppressive, and hypoxia conditions in tumors, all of which can hamper anti-cancer immune responses induced by vaccines [10]. Disruption of immune suppression in tumors through the use immunotherapies in combination with vaccines is, therefore, expected to enhance therapeutic efficacy. Alternatively, induction of anti-cancer immune responses by vaccines may enhance the efficacy of immunotherapeutic agents.

### Tumour antigens for cancer vaccines

**Fig. 2:**
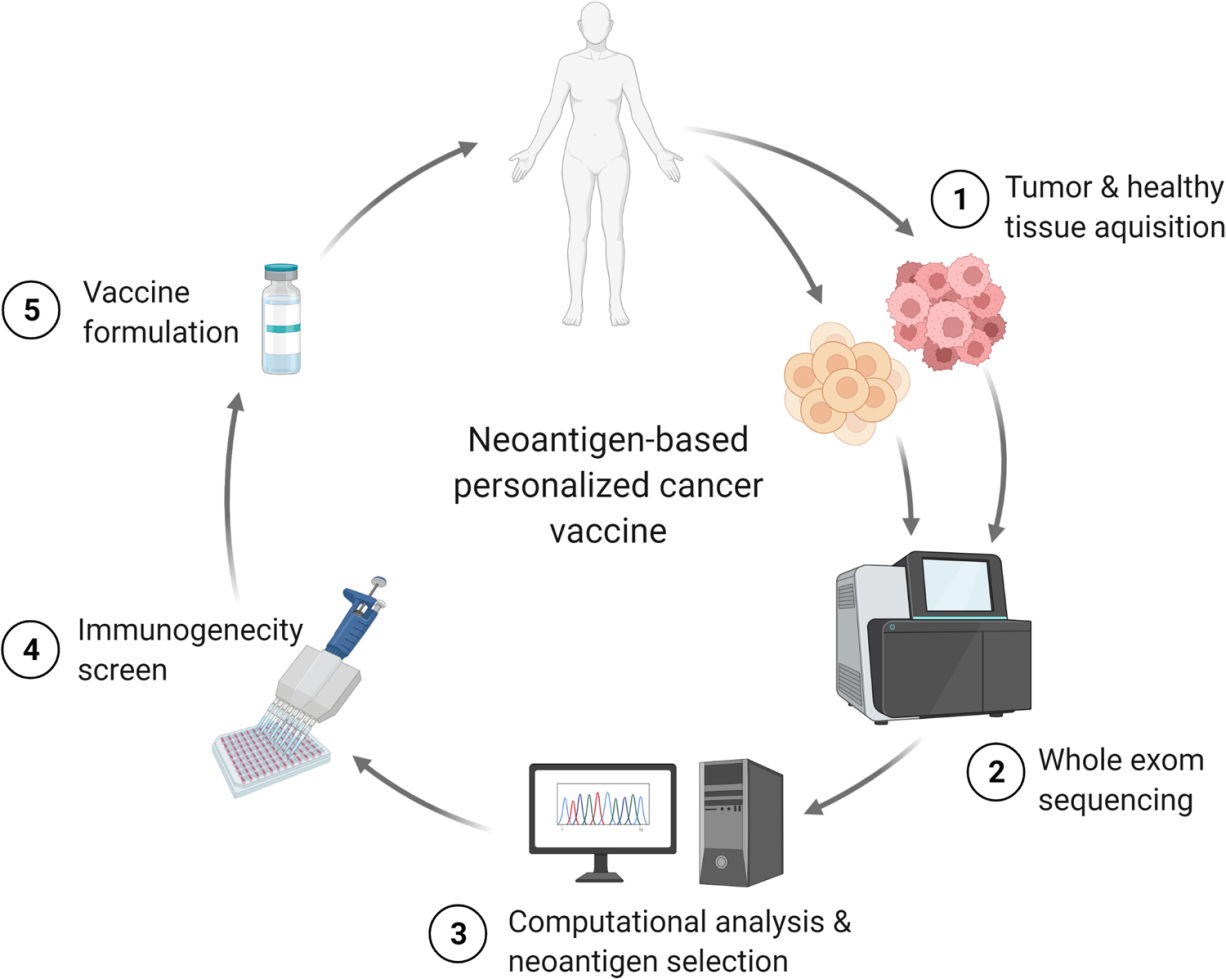
Personalized neoantigen-based cancer vaccine. Neoantigens are recognized by whole exome sequencing of tumour genome and comparing it to the sequences from normal tissue. Identified neoantigens are screened for immunogenicity and used in vaccine preparation to immunize the patient

One of the challenges in developing cancer vaccines has been the nature of the antigens in tumours. An ideal tumour antigen for cancer vaccines would have high expression levels specifically in tumour cells, and broad expression pattern in multiple cancers. Based on their expression profiles, tumour antigens can be of two types: (i) tumour-associated antigens (TAA), and (ii) tumour-specific antigens (TSA) [71]. TAAs are self-antigens that may be expressed in normal tissues but overexpressed in multiple types of cancers (e.g., survivin), expressed in specific tissues and cancers of those tissues (e.g., Melan-A in melanoma and melanocytes), or mainly expressed in cancer tissues and their genes are silenced in normal adult tissues (e.g., cancer-testis antigens) [72–74]. TAA that are highly expressed in several types of tumours but have zero or at least low expression in healthy and mature somatic cells, have the potential to be targeted for cancer vaccines and immunotherapy. A major disadvantage of using TAAs for cancer immunotherapy is that TAAs are self-proteins, and therefore, inducing an immune response against them means breaking self-tolerance against self-antigens, which carries the risk of inducing autoimmunity. TSAs, on the other hand, are mutated self-antigens, also known as neoantigens, which are specific to each tumour, and are expressed on tumour cells only. Neve-rthe-less, there still remains the possible of cross-reactivity with the native antigen, resulting in autoimmune responses against self-antigens. As well, antigen loss may occur in tumours, rendering antigen-specific immune responses against the lost antigen ineffective. Additionally, not all neoantigens are immunogenic, making their identification, screening for immunogencity, and use in the development of personalized cancer vaccines expensive and arduous (Fig. 2) [75].

The discovery of neoantigens have opened new avenues for antigen screening and selection for cancer immunotherapy and vaccines. Gene mutations that occur during carcinogenesis take place in the coding and non-coding regions of the tumour cell’s genome. When a mutation occurs in the coding region of a gene, it results in a change in the amino acid sequence of the protein it codes for, and the expression of a neoantigen in the tumour cell [76]. Neoantigens are highly specific to each individual and are different from the traditional TAA [77]. Because neoantigens are expressed only on tumour cells but not normal cells, they are recognized as non-self antigens by the immune system. Tumour neoantigens are broadly categorized into two types: (i) shared neoantigens, and (ii) personalized neoantigens. Shared neoantigens are similar in different cancer patients; thus, it can be used in a wide-spectrum therapeutic approach for patients with the same mutated gene [78]. On the other hand, personalized neoantigens are unique and differ from patient to patient, therefore, the application of neoantigens in this case should be targeted to each individual (i.e. personalized therapy) [79].

All mutations that occur in tumours: point mutations, inversion, fusion, reading-frame alterations, and insertion-deletions, can generate neoantigens [80–82]. However, not all neoantigens are immunogenic and certain conditions must be met for a neoantigen to induce anti-tumour immunity. These conditions include sufficient expression of the specific neoantigen, high affinity of binding to the patient HLA molecules, and efficient recognition by the patient’s T cells [81]. Indeed, neoantigens with a strong affinity towards HLA molecules, as well as CD4^+^ and CD8^+^ T cells specific to these neoantigens have been detected in cancer patients [83–85].

The first step in neoantigen screening is the comparison of genomic DNA sequences from the normal cells and tumour cells (Fig. 2). This step is complex as some of the tumour’s mutations occur in the non-coding region of the genome and not all mutations are non-synonymous, that is they do not change the amino acid sequences of the protein. Moreover, the identification of mutant proteins that can elicit an anti-tumour immune response can be a challenging process [86]. Current development of bioinformatics tools and algorithms have increased the reliability and accuracy of neoantigen prediction and identification [87]. Next generation sequencing (NGS) has become one of the most versatile techniques that scientists have relied on for neoantigen discovery. Additionally, mass spectrometry (MS) has been used to predict post-translational modifications of neoantigens [88, 89]. In addition to the tumour genome sequencing data, information about the patient’s HLA haplotype, affinity of the mutated peptides towards the patient’s HLA, peptide-HLA complex stability, the affinity between peptide-HLA complex and T cell receptor, and T cell responses against the mutated peptides are needed to ascertain the immunogenicity of neoantigens and the specificity of anti-tumour immune responses [90–92]. It is estimated that only 10% of non-synonymous mutations in tumour cells can generate peptides with high affinity to the patient HLA molecules, and only 1–2% of peptide-HLA complex can stimulate CD8^+^ T cells [93].

Despite the challenges of neoantigen screening and identification, several studies have demonstrated the potential for the use of neoantigens in eliciting anti-tumour immune responses. Castle et al. was the first to use synthetic long peptide (SLP)-based vaccine using neoantigens in mouse melanoma models. From over 50 mutations identified, two mutated antigens showed significant therapeutic effects in these mouse models [87]. In 2015, Carreno et al. reported, for the first time, a personalized DC-based vaccine to treat patients with melanoma. In this study, neoantigens were identified using an immunohistochemistry approach and then patient’s DCs loaded with the neoantigens were transfused into patient, which resulted in enhanced anti-tumour T cell responses [94]. Similarly, Sahin et al. reported the use of RNA-based personalized vaccine using neoantigens identified in a trial that included 13 melanoma patients. Eight of these patients had no further tumour development over the following 23 months [95].

Several obstacles remain in the development and widespread use of neoantigen-based personalized cancer vaccines. The long cycle of cancer genome sequencing, neoantigen identification, and verification of immunogenicity add to the high cost of personalized vaccines, making them economically unfeasible for widespread use in cancer patients [79]. Development of improved bioinformatics tools for characterization of neoantigens, a more in depth understanding of tumour immunology, and advances in vaccine development and delivery methods will prove critical for the development and use of novel neoantigen-based cancer vaccines.

## Modern and emerging vaccine technology

The fundamental goal of all vaccines is to prevent diseases, and their possible devastating consequences [96]. Any specific vaccine works by eliciting an immune response against an antigen that is found in the target pathogen [97]. Once primed by vaccination, the immune system will produce antibodies, cytotoxic cells, and memory cells that can neutralize and destroy the target pathogen before it causes a serious disease [96, 97]. There are many current and emerging types of vaccines that use either protein-based or gene-based approaches [98].

### Nucleic acid vaccines

Two exciting examples of vaccine technologies that are currently in research are the deoxyribonucleic acid (DNA)- and ribonucleic acid (RNA)-based vaccines, which are referred to as genetic vaccines, or most commonly, nucleic acid vaccines [99, 100]. Currently, there are no DNA vaccines approved for human use, and the first RNA-based vaccines were rapidly developed in the wake of the COVID-19 pandemic [101, 102]. RNA-based vaccines use a type of RNA called messenger RNA (mRNA), which carries the genetic information for a protein antigen [98, 103]. mRNA vaccines for COVID-19 have been approved by Health Canada, granted emergency use authorization by the FDA, and granted emergency use validation by the WHO.

Unlike traditional vaccines that use a live attenuated or inactivated pathogen, or parts of the pathogen (subunit vaccines) as the source of the antigen, the nucleic acid vaccines use the genetic code for protein antigens derived from a pathogen to elicit an immune response [104]. Nucleic acid vaccines do not contain weakened or killed versions of the pathogen, or parts of the pathogen as the source of the antigen [102]. Instead, they make use of the cellular processes for protein synthesis to produce a protein, or a peptide, that triggers an immune response against a pathogen in what’s called a gene-based approach [98, 101, 105].

A key challenge in nucleic acid vaccines is shielding them from being degraded before reaching the target site. To avoid degradation of DNA vaccines by DNases and other nucleases, nano-carriers are often used [101]. There are many types of nano-carriers that are being studied, such as: nano-carriers composed of inorganic material, lipid-based nano-carriers, protein-based nano-carriers, and polymeric nano-carriers [101]. mRNA vaccines have been found to be most effective when the mRNA is bound to lipofection formulation [98]. A critical part of mRNA vaccines is successfully transporting the mRNA into the cells [106]. Utilizing lipid nanoparticles (LNP) is the most common method of mRNA vaccine delivery. Liposome-based transfection reagents containing cationic lipids are used to protect the mRNA from degradation by RNases as it makes its way into the cell through the extracellular space [98, 106]. LNPs are designed with an aqueous core, where the mRNA resides, which is enclosed by a lipid bilayer shell [106]. The recent development of ionizable lipids and lipid-like materials has increased the potency of LNPs [106]. Phospholipids, cholesterol, and lipid-anchored polyethylene glycol are also commonly used for both structural and functional purposes in LNPs [106]. Another exciting prospect of LNPs is the potential to embed lipophilic compounds into the lipid bilayer, including adjuvants to increase efficacy of the mRNA vaccine [106]. In addition to LNPs, extracellular exomes, which are key mediators of intercellular communication, are also extensively studied as a method for cancer vaccine delivery. Their optimal size, biocompatibility, stability and target specific delivery makes exomes attractive nano-carriers for vaccines [107].

There are several different routes of administration for DNA vaccines, including injection (Intravenous or Intramuscular), oral, topical, and pulmonary routes [101]. The most common method of delivery for mRNA vaccines is intramuscular injection [106]. This is the route of administration used in all the seven mRNA vaccines for COVID-19 that contain lipid nanoparticles [98]. Intramuscular injections are simple to perform, requiring little personnel training, and mRNA molecules are delivered to the muscle cells for protein synthesis [106]. Other modes of mRNA vaccine delivery that have been investigated include subcutaneous, intradermal, intravenous, intranasal, intranodal, and intraperitoneal methods [106].

### How do nucleic acid vaccines work?

To understand how nucleic acid vaccines work, it is crucial to understand the normal protein synthesis process at the cellular level. Proteins are essential for many cellular functions in our body. Synthesis of proteins using the genetic code in DNA occurs through 2 stages: transcription, and translation [108]. In transcription, the nucleotide sequences in DNA are copied into a complimentary sequence in an mRNA molecule. Transcription occurs in the nucleus where the DNA is stored. The mRNA molecules are then transported into the cytoplasm where translation occurs. During translation, the nucleotide sequence in the mRNA, specifying an amino acid sequence, is read by ribosomes, which are organelles in the cytoplasm. Ribosomes move along the mRNA strand and use the genetic code of the mRNA sequence to translate the codons into their corresponding amino acids, which are then assembled to form a polypeptide and further processed to form mature proteins.

**Fig. 3:**
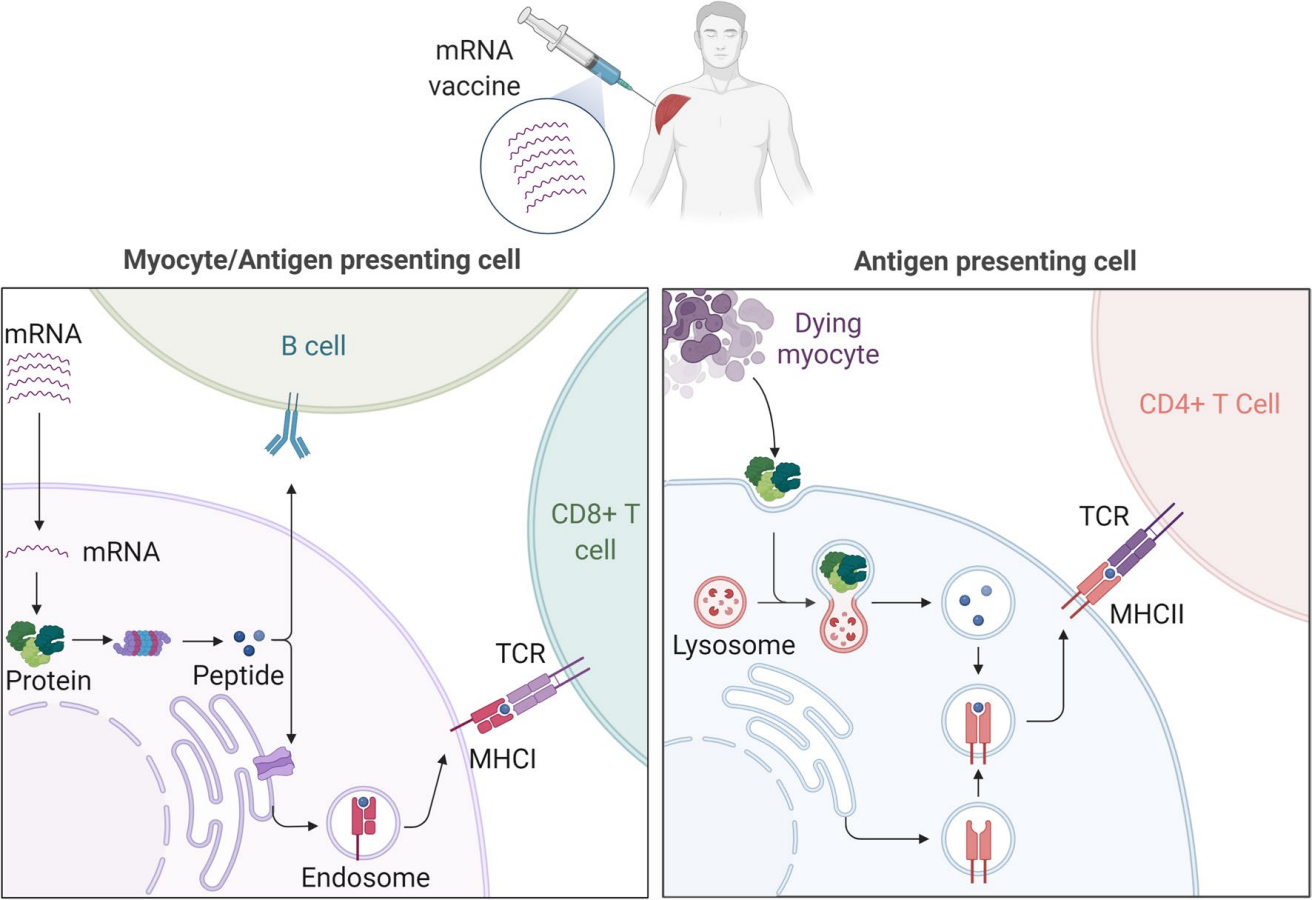
mRNA vaccine. mRNA vaccines work by inducing cells to produce antigens. Once inside the cells, mRNA is translated into proteins, which are processed into antigenic peptides presented on MHC molecules. Antigen presenting cells can also take up proteins released by dying cells to process and present them on MHC molecules. B cells and T cells that can recognize antigenic peptides and peptide:MHC complexes, respectively, are activated to initiate an adaptive immune response

Nucleic acid vaccines use the cellular process of protein synthesis to produce antigenic peptides in the cells that will trigger an antigen-specific immune response. Following a route of injection, nucleic acid molecules (DNA or RNA) must enter keratinocytes and myocytes, as well as antigen-presenting cells (APC) near the injection site, in a process called transfection [101, 109]. DNA vaccines supply the DNA molecule, which contains the genetic code for a protein antigen, to our cells [110]. The DNA in the vaccine is in the form of a circular plasmid, which is derived from bacterial cells [110]. This DNA enters our cells and translocates to the nucleus, where it undergoes transcription to form a mRNA molecule [101]. The mRNA molecules are then translocated back to the cytoplasm, where the genetic code is read by ribosomes to synthesize a protein. mRNA vaccines work through the same cellular processes, however, instead of a DNA molecule it directly provides a pre-synthesized mRNA for translation into an antigenic protein (Fig. 3) [104]. Cellular transfection of myocytes and APCs by the LNP-encapsulated mRNA occur through endocytosis [98]. When the lipid nanoparticle enters the cytoplasm of the cell, they are degraded to release the mRNA in the cytoplasm, where they are translated into proteins by ribosomes [105]. The antigenic proteins are process by the antigen processing and presentation machinery in the cells to generate antigenic peptides, which can be recognized as foreign antigens by B cells directly and by T cells when they are presented on MHC molecules. APCs also take up proteins released from dying myocytes, which are then processed and presented on MHC molecules to activate T cells.

### Advantages of nucleic acid vaccines over traditional vaccines

The benefit of mRNA vaccines lies in the possibilities that they hold for the future of safe and effective vaccination. Live-attenuated vaccines are the most potent type of vaccine in terms of activating both humoral and cellular immune responses [106]. Subunit vaccines are successfully used to generate humoral immunity against pathogens, but they do not generate cellular immunity, and often require the use of adjuvants to boost immunogenicity of the antigens [106]. Generating cellular immunity is critical to destroying the intercellular pathogen reservoir in some chronic diseases [106]. A significant drawback of live-attenuated vaccines is their rare but concerning potential to cause disease in immunosuppressed individuals [106]. Therefore, it is usually recommended that immunosuppressed patients do not receive live-attenuated vaccines [106]. mRNA and DNA vaccines have been developed with the hope of solving both safety and efficacy issues [106]. Nucleic acid vaccines have been proven to induce both cellular and humoral immunity (like live-attenuated vaccines), while also being safe (like subunit vaccines) since they do not contain whole or parts of the pathogen [106, 111]. Another advantage of nucleic acid vaccines, when compared to traditional vaccines, like live-attenuated vaccines and inactivated vaccines, is their ability to be manufactured rapidly [98]. The manufacturing process of the more traditional vaccines have safety concerns and carry a high risk of contamination with live pathogens [99]. The simple and safe manufacturing processes for nucleic acid vaccines are key reasons that they have been extensively researched [99].

Nevertheless, there are still safety concerns with nucleic acid vaccines that must not be overlooked, particularly with DNA vaccines. Since, DNA resides in the nucleus of cells, the DNA molecules in the vaccine must be transported to the nucleus of the cells [102, 106]. To facilitate transport of DNA into the nucleus, viral vectors, such as adenoviruses, are used as vehicles for DNA delivery [112]. However, the use of such delivery vectors could have potential safety concerns collectively referred to as vector-mediated genotoxicity [112, 113]. mRNA vaccines do not have to cross the nuclear membrane and are translated in the cytoplasm. Hence, they do not have similar safety concern as DNA vaccines [106]. In addition, mRNA molecules stay in the cytoplasm of the cell for a relatively short period of time before it is degraded [106].

### Nucleic acid vaccines for cancer

The idea of using nucleic acid vaccines for the treatment of cancer, using both DNA- and RNA-based approaches, is a radical departure from the more traditional methods of cancer treatment, and represents a new era in cancer therapy. Generally, the goal of most vaccines is to prevent someone from either contracting a pathogen, or become seriously ill from the disease it causes, hence are called prophylactic vaccines [114]. Most cancer vaccines being researched are therapeutic in nature, i.e., they are used to treat the patient after they have developed a disease [99]. This is a key difference between the goals of nucleic acid vaccines for pathogens and nucleic acid vaccines for cancers. Therapeutic nucleic acid vaccines for the treatment of cancers rely on similar principles as the preventative vaccines, i.e., to teach the immune system to identify tumours and destroy them [99]. This is done by targeting antigens that are commonly expressed on cancer cells [115]. These antigens include growth associated factors, or antigens that are unique to cancer cells due to somatic mutations (neoantigens) [115]. Both mRNA and DNA vaccines work by delivering the genetic information to cells necessary to produce tumour antigens, and then induce immune responses against these antigens [99]. These antigen-specific immune responses, in particular the cytotoxic T cells, have the potential to clear tumour cells from the body, and therefore aid in cancer treatment (Fig. 3) [115, 116]. In a recent study, whether anti-tumour responses mediated by immune checkpoint inhibitors (ICIs) in solid tumour could be enhanced through immunizing patients against tumour antigens using mRNA vaccines was studied [117]. In the Phase I of the trials sponsored by ModernaTX Inc., mRNA-4157 was used as monotherapy in patients with resected solid tumours and in combination with pembrolizumab in patients with unresectable solid tumors. This combination was found to be safe and it induced neoantigen specific T cell responses in the immunized patients [117]. Currently, phase II trial is in progress (NCT03739931).

## Lessons learned from the COVID-19 vaccines

COVID-19 is the disease caused by the virus SARS-CoV-2[98, 118]. SARS-CoV-2 is an enveloped virus with a positive-sense single-stranded RNA genome, belonging to the β-coronavirus subfamily [98]. An important part of the SARS-CoV-2 virus is it’s ‘spike (S) protein, which is a relatively large surface glycoprotein containing 1300 amino acids [98, 105]. It is encoded by the virus’ 3,822-bp *S* gene [98]. The role of the S protein is to interact with the host cells by binding to angiotensin-converting enzyme 2 (ACE2), allowing the virus to fuse with the cell membrane and enter cells [119, 120]. COVID-19 has a profound impact on certain organs, such as the lungs, heart, and kidneys, likely due to high expression of ACE2 on epithelial cells in these organs [121].

In response to the COVID-19 pandemic, many vaccines were developed. Safe and effective vaccines against SARS-CoV-2 are viewed as essential to conquering the COVID-19 pandemic, which has had an exceptionally negative impact on physical and mental health. Currently, there are over 100 vaccine candidates for SARS-CoV-2 being studied around the world according to the WHO. The leaders in the race to develop a vaccine against SARS-CoV-2 emerged as two mRNA vaccines, made by Pfizer and BioNTech, and by Moderna. These vaccines were both developed in under a year, which is considered incredible, since the fastest prior vaccine brought to the market was made in approximately 4 years. Along with the two mRNA vaccines, some of the other fastest developed vaccines against SARS-CoV-2 use viral vector technology, such as those developed by AstraZeneca [122].

Conventional vaccines usually take years or decades to manufacture and test, and ultimately obtain approval for human use [105]. Typically, they are very expensive to produce [105]. This makes them ill-suited to combat global pandemic from novel viruses, like SARS-CoV-2. mRNA vaccines however have the advantage of relatively low cost and rapid manufacturing.

To accelerate the process of approval, there were numerous strategies employed. In the United States, the FDA granted three vaccines developed by Pfizer-BioNTech, Moderna, and Janssen an Emergency Use Authorization (EUA). According to the FDA, an EUA is granted when the known and potential benefits outweigh the known and potential risks of the vaccine [123]. Granting an EUA to these vaccines allows them to be rolled out much faster than having to wait for a full FDA approval, which takes longer to achieve. As of August 23, 2021, the FDA has given full approval to the Pfizer-BioNTech COVID-19 Vaccine [123].

A critical problem in the effort to vaccinate the world against COVID-19 comes from widespread vaccine hesitancy and/or skepticism. As of early August 2021, the US COVID-19 vaccine rollout has slowed to concerning levels, with a substantial portion of the population refusing to be vaccinated against COVID-19. This is often attributed to a rise in misinformation due to the development of social media platforms with several conspiracy theories, specifically regarding the mRNA technology. Some of the conspiracy theories include belief of impaired fertility following vaccination, fear of mRNA integrating itself into the human genome and altering our DNA, and several others related to religious and political views. While none of these conspiracy theories are merit based, they persist and circulate in populations around the world, including the United States and Canada where vaccines are widely accessible [124].

Another challenge for some mRNA vaccines is the requirement for storage and transport at ultra-low temperatures [98]. One possible avenue for combatting this challenge of mRNA vaccines is developing new types of lipid nanoparticle technologies that are more stable at ambient temperature [98]. These lessons learnt and experiences gained from developing mRNA vaccines and immunizing nations against COVID-19 are going to be valuable in developing mRNA vaccines against cancer [125].

## Conclusion

Cancer Immunotherapy properly named as the “Breakthrough of the year in 2013” has revolutionized the treatment of several types of cancer. The tremendous experience with nucleic acid vaccines in the COVID-19 era, will move the file of cancer vaccine forward. A time may come when cancer vaccines will be part of the immunization history, and oncologists share the joy of a pediatrician knowing that immunized patients are protected not only against infectious pathogens but against specific types of cancers [125].
